# Decline in Mental Health in the Beginning of the COVID-19 Outbreak Among European Older Adults—Associations With Social Factors, Infection Rates, and Government Response

**DOI:** 10.3389/fpubh.2022.844560

**Published:** 2022-03-14

**Authors:** Daniel Lüdecke, Olaf von dem Knesebeck

**Affiliations:** Institute of Medical Sociology, University Medical Center Hamburg-Eppendorf, Hamburg, Germany

**Keywords:** COVID-19, mental health, social factors, elder people, NPI, depression, anxiety

## Abstract

**Objective:**

Governments across the world have deployed a wide range of non-pharmaceutical interventions (NPI) to mitigate the spread of COVID-19. Certain NPIs, like limiting social contacts or lockdowns, had negative consequences for mental health in the population. Especially elder people are prone to mental illnesses during the current pandemic. This article investigates how social factors, infections rates, and stringency of NPIs are associated with a decline in mental health in different European countries.

**Methods:**

Data stem from the eighth wave of the SHARE survey. Additional data sources were used to build macro indicators for infection rates and NPIs. Two subsamples of persons with mental health problems were selected (people who reported being depressed, *n* = 9.240 or nervous/anxious, *n* = 10.551). Decline in mental health was assessed by asking whether depressive symptoms or nervousness/anxiety have become worse since the beginning of the COVID-19 outbreak. For each outcome, logistic regression models with survey-design were used to estimate odds ratios (OR), using social factors (age, gender, education, living alone, and personal contacts) and macro indicators (stringency of NPIs and infection rates) as predictors.

**Results:**

Higher age was associated with a lower likelihood of becoming more depressed (OR 0.87) or nervous/anxious (OR 0.88), while female gender increased the odds of a decline in mental health (OR 1.53 for being more depressed; OR 1.57 for being more nervous/anxious). Higher education was only associated with becoming more nervous/anxious (OR 1.59), while living alone or rare personal contacts were not statistically significant. People from countries with higher infection rates were more likely to become more depressed (OR 3.31) or nervous/anxious (OR 4.12), while stringency of NPIs showed inconsistent associations.

**Conclusion:**

A majority of European older adults showed a decline in mental health since the beginning of the COVID-19 outbreak. This is especially true in countries with high prevalence rates of COVID-19. Among older European adults, age seems to be a protective factor for a decline in mental health while female gender apparently is a risk factor. Moreover, although NPIs are an essential preventative mechanism to reduce the pandemic spread, they might influence the vulnerability for elderly people suffering from mental health problems.

## Introduction

The new coronavirus infection (COVID-19), first discovered in December 2019 in Wuhan City, China, has spread worldwide. Accordingly, the World Health Organization (WHO) officially declared the outbreak as international public health emergency (1). COVID-19 is a severe respiratory infection with high infection rate and relatively high mortality, in particular when medication or vaccination was not available. Consequently, governments across the world have deployed a wide range of non-pharmaceutical interventions (NPIs) to mitigate the spread of COVID-19. The responses to COVID-19 in different countries varied from herd immunity strategies, which implied only few or almost no measures, to more assertive approaches based on the implementation of a wide range of stringent NPIs (2, 3). The prevalence of COVID-19 affected the stringency of NPIs implemented in different countries. However, countries undertaking more strict measures were also able to contain the pandemic outbreak. Thus, there were both great variations in the stringency of government responses and infection rates of COVID-19 between countries worldwide (4, 5).

One of the main goals of the various NPIs was to protect risk groups who are particularly vulnerable to COVID-19. Especially old age was highlighted early as a risk factor for adverse outcomes of a COVID-19 infection and is one of the most important determinants of mortality (6, 7). Studies suggest that the odds of in-hospital deaths increase by a factor of 1.1 per year of age (8). The downside of certain NPIs, such as limiting social contacts, gathering restrictions, or short- and medium-term lockdowns, is an increased risk of negative consequences for mental health in the population. Again, especially the older population is a risk group that is prone to mental illnesses during the current pandemic (9, 10).

Mental health is not only affected by limited social contacts and lockdowns. Rather, widespread occurrences of infectious diseases in general, and of COVID-19 in particular, are closely related to symptoms of psychological distress and affected mental health (11). In a survey among the Chinese population, 37.1% of the elderly had experienced depression and anxiety during the pandemic (12, 13). Studies about previous experiences with outbreaks of infectious diseases also have found that the proportion of people who are mentally affected by a pandemic is higher than people who are physically infected. In this regard, depressive feelings, anxiety and fear were common adverse effects (14, 15).

It is known that social factors such as social contacts and relationships, serve as protective buffers that lower the risk of morbidity and mortality (16). Furthermore, social networks have a positive impact on mental health (17). However, the psychological distress for the general population caused by the COVID-19 pandemic and its government response measures is not fully understood yet. International studies mostly focus on the mental health impact of infected quarantined patients and rarely on government response measures in general (18).

Against this background, this article addresses the following research questions: (1) What is the prevalence of a decline in mental health in different European countries during the COVID-19 pandemic? (2) What are the associations of social factors (gender, age, education, and social contacts) with a decline in mental health? (3) Do the associations vary according to infection rates and government response measures?

## Materials and Methods

### Sample and Participants

Analyses were based on data from the eighth wave of SHARE, the Survey of Health, Aging, and Retirement in Europe (19). SHARE is a large pan-European social science panel study. The first wave of data collection started in 2004, including 12 countries. New waves of data collection were repeated every 2–3 years. Refresher samples were drawn to compensate for panel attrition. In total, about 530,000 interviews with people from 28 European countries and Israel were conducted. Topics covered in the interviews are, among others, social networks, physical and mental health, employment, retirement, financial situation, and social activities. The wave of 2020 was the first one that included data on the pandemic outbreak and was carried out in 27 countries. Based on population registers, SHARE used probability samples within the countries and includes non-institutionalized adults aged 50 years or older and, if available, their partners. Exclusion criteria were: being incarcerated, moved abroad, unable to speak the language of questionnaire, deceased, hospitalized, moved to an unknown address or not residing at sampled address (20, 21). The outbreak of the COVID-19 pandemic hit the data collection in the middle of the 8th wave, thus fieldwork was suspended and a specific COVID-19 questionnaire was developed to collect data with pandemic related topics. The fieldwork for this survey started in June 2020 and ended in September 2020.

The final SHARE sample consisted of *N* = 46.500 participants from 27 countries. As we were interested in the decline of mental health, we only considered those respondents who previously reported being depressed and/or being nervous or anxious (see measures). Thus, for the present study, two subsamples of persons with mental health problems were selected (people who reported being depressed, *n* = 9.240 or nervous/anxious, *n* = 10.551).

### Additional Data Sources

Additional data sources were used to build two macro indicators. The stringency in government response toward the COVID-19 outbreak was based on the Oxford COVID-19 Government Response Tracker (OxCGRT), a global panel database of pandemic policies that covered more than 180 countries (22). For each country at each day of the pandemic, measures to reduce the spread of the outbreak, like longer or stricter lockdown measures, reduced social contacts, and similar, were recorded and a daily index of government response for each country (stringency index) was calculated. Countries with higher stringency index have undertaken more intensive measures to reduce the spread of COVID-19. Data was filtered to include only those countries that were also present in this survey.

Data on numbers of confirmed cases of people infected with COVID-19 were taken from the COVID-19 Data Repository by the Center for Systems Science and Engineering (CSSE) at the Johns Hopkins University (23). Data on confirmed cases are collected from more than 190 countries. Based on these data, the percentage of the population infected with COVID-19 was calculated. Again, only data from countries that were also included in this survey was used.

Both data sources were continuously updated and thereby reflected the evolution of infection rates and government measures during the pandemic. To adequately represent the situation in the participants' countries at the time of the SHARE fieldwork, only those data for government response and confirmed cases that referred to the period from the beginning of the COVID-19 outbreak until the time of field work in each SHARE country was used (i.e., summer 2020).

### Measures

#### Dependent Variables

Mental health problems were assessed by asking “In the last month, have you been sad or depressed?.” If answered yes, a person is considered as “depressed.” Similarly, people were considered as being nervous or anxious if they answered “yes” to the question “In the last month, have you felt nervous, anxious, or on edge?.” Decline in mental health was then assessed by asking those respondents who were depressed or nervous/anxious, whether that has “been more so, less so, or about the same as before the outbreak of Corona?.” The variable was dichotomized. The option “more so” was considered as decline in mental health, “less so” and “about the same” refers to no decline in mental health. Both mental health indicators were recoded this way.

#### Independent Variables

Social factors included in the regression models are respondent's age, gender, education, whether they lived alone in their household and personal contacts. Education was based on the International Standard Classification of Education (24), which ranges from 0 to 6 (low to higher education) and is recoded into three levels: “low (lower/upper secondary),” “mid (post-secondary),” and “high (tertiary).” Personal contacts describe the intensity of contacts that respondents had with family and friends from outside their home since the outbreak of COVID-19. The question was “Since the outbreak of Corona, how often did you have personal contact, that is, face to face, with the following people from outside your home? Was it daily, several times a week, about once a week, less often, or never?.” People were asked to respond to this question with regard to their (a) children, (b) parents, (c) other relatives, and (d) neighbors, friends, or colleagues. Rare personal contacts are present if the respondents had personal contacts less than once a week in relation to all of the four groups.

The stringency in government response toward the COVID-19 outbreak and the percentage of the population infected with COVID-19 were used as macro indicators in the regression models. For each country, the mean value for the daily Oxford stringency index values, from the start of the pandemic until the beginning of the fieldwork, was calculated. This variable represents the average government response within each country to the COVID-19 outbreak and has a possible range from 0 to 100 (22). Based on the distribution of this variable, an indicator was built, with countries being classified into three groups: low stringency (average Oxford stringency index <40), middle stringency (index between 40 and 45) and high stringency (index > 45). The proportion of the population infected with COVID-19, which was calculated based on the data from the COVID-19 Data Repository, refers to the cumulative percentage from the beginning of the pandemic until the start of the SHARE data collection in each country (i.e., summer 2020).

### Statistical Analysis

Descriptive statistics were used to document the sample characteristics. For each of the two dependent variables indicating decline in mental health, three logistic regression models for complex samples were calculated, using quasi-binomial links to properly account for survey-weighting, disproportional sampling, and selective mortality. The country variable was used to define the strata in the survey-design, hence the regression models accounted for the fact that respondents were clustered within different countries. Robust Horvitz-Thompson standard errors are reported (25). The first model only included social factors, the second model only included the two macro indicators, and the third (full) model included both social factors and macro indicators. Models were checked for the presence of multicollinearity. All models had a variance inflation factor (VIF) below 2.8, indicating no severe collinearity issues (26). All analyses were performed using the R statistical package (27), including the packages “survey” (28), “ggplot2” (29), and “parameters” (30).

## Results

### Classification of Stringency and COVID Infection Rates

Each of the country classifications “low stringency,” “middle stringency,” and “high stringency” included nine countries (see Table 1). The average stringency index for low stringency countries varied from 34 to 39. Latvia, Slovenia and Bulgaria had rather low COVID-19 infection rates of 0.06, 0.07, and 0.08%, respectively. Countries with a middle stringency had a mean stringency index from 40 to 44. Overall, these countries showed the lowest COVID-19 infection rates from all three stringency classifications. High stringency countries had an average stringency index ranging from 45 to 56. The proportion of people infected with COVID-19 ranged from 0.11 to 0.60%, and overall, these countries had the highest infection rates.

**Table 1:** Average stringency index (SI) and percentage of COVID infections in the population by country, based on data from the Oxford COVID-19 Government Response Tracker and the COVID-19 Data Repository from CSSE.

**Low stringency countries (SI** **<** **40)**			**Middle stringency countries (SI 40–45)**			**High stringency countries (SI** **>** **45)**		
**Country**	**Average SI**	**COVID infections, %**	**Country**	**Average SI**	**COVID infections, %**	**Country**	**Average SI**	**COVID infections, %**
Bulgaria	38	0.08	Austria	41	0.22	Belgium	45	0.52
Denmark	39	0.21	Czech Republic	40	0.09	Croatia	47	0.60
Estonia	35	0.15	Germany	43	0.23	Cyprus	47	0.11
Finland	37	0.13	Greece	44	0.03	France	51	0.30
Latvia	38	0.06	Hungary	41	0.04	Israel	50	0.19
Luxembourg	38	0.66	Lithuania	43	0.06	Italy	56	0.39
Slovenia	39	0.07	Netherlands	43	0.29	Malta	47	0.12
Sweden	34	0.53	Poland	44	0.07	Romania	46	0.11
Bulgaria	38	0.36	Slovakia	42	0.03	Spain	47	0.52

### Sample Description

Characteristics of the subsample of respondents who reported to be depressed or sad (*n* = 9,240) is shown in Table 2. The mean age of respondents was 72.3 years. 19.3% had high education. 70.0% were female. 36.5% of the participants mentioned that they had rare personal contacts. A share of 32.3% were living alone. 63.0% reported that they felt more depressed since the beginning of COVID-19.

**Table 2:** Sample description of respondents reporting depressive symptoms, SHARE data (8th wave), unweighted.

**Country**	***N***	**Mean age (SD)**	**High education, %**	**Female gender, %**	**Rare personal contacts, %**	**Living alone, %**	**More depressed, %**
**Low stringency countries (SI** **<** **40)**							
Bulgaria	174	71.2 (9.1)	14.9	69.0	20.8	35.1	58.0
Denmark	203	71.7 (9.5)	42.3	70.9	33.0	37.4	62.6
Estonia	691	74.6 (9.7)	21.4	74.4	42.2	43.7	61.2
Finland	210	71.1 (9.7)	35.7	69.0	39.8	26.2	59.5
Latvia	137	71.6 (9.8)	24.8	73.0	45.9	47.4	45.3
Luxembourg	208	70.2 (8.5)	17.5	66.3	45.2	22.6	72.1
Slovenia	412	73.6 (9.3)	13.6	70.6	34.5	26.2	52.4
Sweden	221	75.8 (8.0)	38.2	65.6	25.7	41.6	69.7
Switzerland	326	73.5 (9.4)	17.8	70.9	46.6	38.3	70.6
Total	2,582	73.1 (9.4)	23.3	70.8	38.2	36.1	61.5
**Middle stringency countries (SI 40–45)**							
Austria	316	75.3 (9.2)	26.0	73.1	33.5	42.4	52.8
Czech Republic	418	73.4 (8.0)	12.9	76.1	30.7	39.7	50.5
Germany	626	71.4 (9.5)	30.9	66.0	29.9	33.5	53.2
Greece	719	72.2 (10.1)	13.8	68.3	30.6	34.4	74.0
Hungary	167	72.2 (7.6)	17.2	70.7	26.9	39.5	41.9
Lithuania	327	71.9 (10.3)	33.6	70.6	50.8	36.4	63.0
Netherlands	108	72.4 (9.1)	36.7	74.1	37.4	49.1	71.3
Poland	613	69.4 (9.4)	11.9	67.2	34.0	21.7	42.6
Slovakia	192	66.3 (8.6)	5.2	64.1	35.1	22.9	49.5
Total	3,486	71.6 (9.5)	19.8	69.3	33.5	33.6	56.0
**High stringency countries (SI** **>** **45)**							
Belgium	439	71.2 (9.6)	35.3	72.0	36.2	40.8	82.5
Croatia	296	71.0 (8.9)	11.1	67.2	28.6	24.3	56.4
Cyprus	92	75.0 (9.6)	7.6	79.3	56.0	37.0	69.6
France	529	73.7 (9.8)	28.2	73.9	34.9	41.2	67.5
Israel	164	74.6 (8.8)	26.4	68.9	41.5	34.8	73.2
Italy	722	72.2 (9.5)	7.2	68.1	39.6	19.3	77.0
Malta	212	70.5 (8.9)	2.8	66.5	69.8	17.9	78.8
Romania	326	69.9 (9.6)	3.4	69.3	42.9	21.5	58.6
Spain	392	75.2 (9.0)	9.9	70.4	23.7	19.6	76.0
Total	3,172	72.4 (9.5)	15.6	70.2	38.3	27.9	71.9
**All countries**							
Total	9,240	72.3 (9.5)	19.3	70.0	36.5	32.3	63.0

Table 3 shows the characteristics of people who reported to be nervous and/or anxious. Their average age was 71.3 years. 37.9% were higher educated and 67.1% were of female gender. Rare personal contacts were reported by 37.8% of the respondents. 26.5% were living alone. 71.6% mentioned being more nervous and/or anxious since the pandemic outbreak.

**Table 3:** Sample description of respondents reporting being nervous and/or anxious, SHARE data (8th wave), unweighted.

**Country**	***N***	**Mean age (SD)**	**High education, %**	**Female gender, %**	**Rare personal contacts, %**	**Living alone, %**	**More nervous/anxious, %**
**Low stringency countries (SI** **<** **40)**							
Bulgaria	241	70.5 (9.3)	14.1	63.9	20.4	29.0	53.1
Denmark	398	69.5 (8.7)	49.4	65.6	41.5	24.4	81.4
Estonia	813	72.6 (9.2)	23.6	69.6	42.2	35.2	69.6
Finland	268	69.3 (9.0)	47.4	64.6	37.1	23.9	60.4
Latvia	215	70.5 (9.5)	22.3	73.0	45.5	34.9	67.4
Luxembourg	239	68.8 (8.0)	18.3	62.8	43.5	20.1	80.3
Slovenia	529	72.4 (9.3)	17.0	69.9	38.4	23.4	66.0
Sweden	267	74.0 (8.5)	42.5	67.0	25.9	36.7	76.4
Switzerland	302	72.1 (9.0)	18.9	71.5	51.0	30.5	80.5
Total	3,272	71.4 (9.1)	27.6	68.0	39.3	29.2	70.7
**Middle stringency countries (SI 40-45)**							
Austria	285	74.3 (9.1)	22.6	69.5	34.0	31.2	67.4
Czech Republic	432	73.2 (7.9)	14.7	75.2	29.6	34.5	66.9
Germany	531	69.9 (9.2)	32.6	65.5	31.5	26.7	71.8
Greece	992	71.6 (10.0)	17.5	64.7	37.0	28.3	83.3
Hungary	169	71.6 (7.2)	17.6	67.5	29.2	31.4	45.6
Lithuania	413	70.3 (10.3)	34.4	71.4	51.1	32.0	74.3
Netherlands	104	71.9 (8.7)	23.3	71.2	35.6	31.7	86.5
Poland	509	69.0 (9.1)	12.2	63.5	36.8	16.5	49.7
Slovakia	182	64.7 (8.2)	6.0	56.6	32.4	15.9	55.5
Total	3,617	70.9 (9.5)	20.6	67.0	36.0	27.4	69.6
**High stringency countries (SI** **>** **45)**							
Belgium	536	70.5 (9.6)	36.1	67.9	36.6	36.0	84.1
Croatia	342	70.0 (8.5)	15.3	63.5	32.1	19.0	64.3
Cyprus	109	73.1 (9.7)	12.8	75.2	54.6	26.6	65.1
France	575	72.7 (9.6)	25.9	72.7	33.3	36.3	73.6
Israel	213	74.4 (8.6)	29.1	70.4	39.8	28.2	72.3
Italy	767	71.1 (9.5)	6.6	61.8	39.3	14.6	77.7
Malta	332	69.2 (8.7)	4.5	63.0	64.8	14.8	78.0
Romania	347	68.3 (8.9)	2.3	61.1	39.8	15.3	61.4
Spain	441	75.3 (8.9)	9.5	69.6	24.0	18.6	77.3
Total	3,662	71.5 (9.4)	15.9	66.4	38.3	23.3	74.5
**All countries**							
Total	10,551	71.3 (9.4)	37.9	67.1	37.8	26.5	71.6

### Factors Associated With Feeling More Depressed Since the COVID-19 Outbreak

Looking at the regression model with social factors included as predictors (model 1, see Table 4), higher age was associated with a lower likelihood of becoming more depressed (OR 0.87). Female respondents were more likely to feel more depressed since the outbreak (OR 1.50). Middle or higher educated persons, compared to people with low educational level, were less likely to feel more depressed (OR 0.61), although only middle education was statistically significant. People who lived alone in their household had a lower chance of feeling more depressed (OR 0.79). Rare personal contacts were not significantly associated with feeling more depressed. Regarding the model with macro indicators (model 2), in comparison to countries with low stringency index, respondents from countries with middle stringency index were less likely to feel more depressive after the COVID-19 outbreak (OR 0.66). The likelihood of feeling more depressed for people from countries with high stringency index, however, was higher in comparison to the low stringency countries (OR 1.37). An increased rate of people infected with COVID-19 was also associated with a higher chance of feeling more depressed (OR 3.24). In the full model (model 3), in which both social factors and macro indicators were included, most associations remained very similar compared to the first two models. The most noticeable change was related to the association with education, which has diminished in model 3.

**Table 4:** Feeling more depressed since pandemic outbreak, survey-weighted logistic regression models, showing odds ratios (OR), 95% confidence intervals (CI), and significances (*p*).

**Predictors**	**Model 1**			**Model 2**			**Model 3**		
	**OR**	**CI**	***P***	**OR**	**CI**	***P***	**OR**	**CI**	***P***
Age	0.87	0.78–0.97	0.011				0.87	0.78–0.97	0.012
Female gender	1.50	1.16–1.95	0.002				1.53	1.17–1.99	0.002
Education: low (ref.)	1.00	–	–				1.00	–	–
Education: middle	0.61	0.46–0.81	0.001				0.96	0.73–1.26	0.750
Education: high	0.73	0.52–1.01	0.058				1.01	0.72–1.41	0.973
Living alone	0.79	0.64–0.98	0.033				0.84	0.69–1.03	0.094
Rare personal contacts	1.06	0.82–1.37	0.640				0.96	0.73–1.27	0.796
SI: low (ref.)				1.00	–	–	1.00	–	–
SI: middle^a^				0.66	0.53–0.82	<0.001	0.64	0.51–0.79	<0.001
SI: high^b^				1.37	1.10–1.71	0.005	1.32	1.05–1.66	0.019
% COVID-19 cases				3.24	1.61–6.49	0.001	3.31	1.66–6.59	0.001
Observations	9,017			9,017			9,017		

a *Middle stringency countries (reference: low stringency countries)*.

b *High stringency countries (reference: low stringency countries)*.

### Factors Associated With Feeling More Nervous and/or Anxious Since the COVID-19 Outbreak

In model 1 (see Table 5), feeling more nervous/anxious was less likely when respondents were older (OR 0.87). Female gender was associated with a higher likelihood of feeling more nervous/anxious (OR 1.54). The probability of feeling more nervous/anxious is higher for people with high education as compared to people with low education (OR 1.34). No statistically significant associations were found for middle education (OR 1.08), living alone (OR 0.99), and rare personal contacts (OR 1.07). While the stringency index was not significantly associated with feeling more nervous/anxious (model 2), the rate of people infected with COVID-19 significantly increased the chance of feeling more nervous/anxious (OR 3.95). In model 3, most associations remained similar compared to the first two models, with an exception for education. The likelihood of feeling more nervous/anxious increased with medium and higher education.

**Table 5:** Feeling more nervous/anxious since pandemic outbreak, survey-weighted logistic regression models, showing odds ratios (OR), 95% confidence intervals (CI), and significances (*p*).

**Predictors**	**Model 1**			**Model 2**			**Model 3**		
	**OR**	**CI**	***P***	**OR**	**CI**	***P***	**OR**	**CI**	***P***
Age	0.87	0.78–0.97	0.015				0.88	0.79–0.98	0.025
Female gender	1.54	1.20–1.99	0.001				1.57	1.21–2.02	0.001
Education: low (ref.)	1.00	–	–				1.00	–	–
Education: middle	1.08	0.81–1.43	0.615				1.37	1.03–1.80	0.028
Education: high	1.34	0.97–1.84	0.077				1.59	1.15–2.19	0.005
Living alone	0.99	0.79–1.23	0.926				1.01	0.81–1.26	0.929
Rare personal contacts	1.07	0.82–1.40	0.608				1.06	0.80–1.41	0.689
SI: low (ref.)				1.00	–	–	1.00	–	–
SI: middle^a^				1.05	0.86–1.29	0.622	1.06	0.86–1.30	0.593
SI: high^b^				0.96	0.78–1.19	0.730	1.10	0.87–1.38	0.434
% COVID-19 cases				3.95	2.01–7.74	<0.001	4.12	2.15–7.91	<0.001
Observations	10,306			10,306			10,306		

a *Middle stringency countries (reference: low stringency countries)*.

b *High stringency countries (reference: low stringency countries)*.

## Discussion

The present study sought to describe the prevalence of decline in mental health among elderly persons in Europe during the COVID-19 pandemic and to analyse the associations of social factors, infection rates and government response measures with a decline in mental health.

### Summary of Main Findings

One finding was that the majority of older adults, who reported depressive symptoms or being nervous and/or anxious, showed a decline in mental health since the beginning of the COVID-19 pandemic. This holds true for respondents across all European countries. Although the proportion of people with a decline in mental health was higher in countries where the social life was affected by strict government response measures, even in countries with a milder response a majority of respondents reported an increase in depressive symptoms, nervousness, and anxiety. Regarding social factors, we found that increasing age and female gender were associated with a lower chance of a decline in mental health, while we found inconsistent or no associations for education, rare personal contacts and living alone. In terms of the macro indicators, there were positive associations with the proportion of infections in the population, but inconsistent results related to the stringency index.

### Interpretation

Although the concrete impact of the COVID-19 pandemic on mental health is not yet fully understood, studies from former comparable outbreaks that required social restrictions or lockdown measures confirmed that the challenges and stress associated with such NPIs could trigger mental disorders, especially anxiety and depressive symptoms (11, 31). This included both, an increase in the prevalence of disorders as well as worsening mental health (18). Beyond the impact of NPIs on mental health, the fear of serious health consequences might exert a further negative influence on mental well-being. The fact that especially older people were at higher risk for adverse health outcomes due to COVID-19 might explain why—compared to surveys that also included younger populations—we found a higher proportion of decline in mental health, which did not only occur in countries with strict government response measures. This is in line with studies that showed the negative psychological impact of the pandemic on the older population due to increased morbidity and mortality risks (13, 32). In our study, we used infection rates of a population and the stringency index as two indicators on a macro level that reflect the pandemic threat and the rigidity of NPIs and found links between those indicators and a decline in mental health. Populations' infection rates were strongly positively associated with feeling more depressed and more nervous/anxious. This can be explained with the health concerns of elderly people, who were a particular risk group for adverse COVID-19 related health outcomes. The perceived threat of the pandemic increased with higher proportions of infected cases in the population. This fear was in particular present in risk groups such as older people (33). We found no evidence that the government response measures, indicated by the stringency index, were associated with feeling more nervous and/or anxious. Feeling more depressed, however, was more likely for respondents living in countries with higher stringency index. Studies that analyzed the impact of lockdown measures and comparable NPIs also reported negative mental health consequences. A longitudinal study from South Africa showed that the prevalence of depression and anxiety symptoms was higher during the time of social restrictions (34). Another study from Israel indicated that such measures were the main risk factor for depression and anxiety (35).

According to our results, increasing age was associated with a lower chance of a decline in mental health. This seemed to be somewhat counter intuitive given that in particular elderly people were one of the most vulnerable risk groups related to COVID-19 and their emotional response toward the pandemic outbreak was more apparent than in younger age groups (13, 36). Research on this topic revealed inconclusive results. While a documentary analysis indicated that mental health in older people was negatively affected by social restriction measures, another recent study found that adherence to such measures was not associated with a decline in mental health. One explanation might be that a key issue that serves as protective factor could be the social connectedness whose positive impact was rated higher than the negative impact of COVID-19 related worries (37, 38).

A clear association was found between gender and decline in mental health, with female persons being more likely to be affected. This is in line with previous research that described significantly higher scores of psychological distress for female gender during the pandemic (13). One study reported significantly larger increase in depressive symptoms for female persons (37). A meta-analysis on the prevalence of depression and anxiety among COVID-19 patients also showed higher proportions of female patients being affected. However, the total effect of gender was not statistically significant (39).

The associations of education and decline in mental health differed between the two subgroups of respondents who were more depressed and those who felt more nervous and/or anxious. In the models with social factors only, higher education showed weak evidence for a lower probability of decline in mental health. In the full model, the association between education and feeling more depressed diminished, while we found a significant positive association between higher education and feeling more nervous and/or anxious. The latter result was supported by other research results. Recent longitudinal studies on the change of mental health in the course of COVID-19 showed that a higher educational level was associated with increases in mental health problems (40). A reason might be that higher educated persons may feel greater concerns about the consequences of COVID-19, which was also reported in a study from the US (41).

Regarding social relations, living alone was associated with feeling more sad or depressed, but this association diminished after controlling for the macro indicators. We did not find clear associations for living alone and feeling more nervous and/or anxious. Rare personal contacts were not statistically significant in any model. Although studies showed a positive impact of social contacts on mental health (17), other findings suggested that there was no clear link between social contacts and nervousness and anxiety for older individuals (42). An explanation for the inconsistent associations between social relations and decline in mental health could be that older persons experience less life changes due to the pandemic, making social contacts more important for younger generations (43).

### Limitations

Some methodological limitations have to be considered that affect the interpretation of the findings. First, the SHARE data only provides some rather crude measures of decline in mental. An advantage of the mental health indicators we used is that they refer to acute and very recent mental health problems. On the other hand, it depends on a subjective perception of feeling depressed, nervous or anxious and is not measured by a validated assessment instrument for mental health disorders. Furthermore, the mental health indicators we used may only reflect a temporary condition and no longer lasting disorder. Nevertheless, we decided to use this indicator as it allowed us to measure a decline of mental health problems since the COVID-19 outbreak using cross-sectional data.

Another limitation is related to the measures of social relations, which might explain the inconsistent or unclear associations in our results. Only frequency of personal contacts, not telephone or digital contacts were assessed. Additionally, as rare personal contacts and living alone only refer to the quantitative dimension of social relations, they do not reflect qualitative or functional aspects. Furthermore, due to the age range in the sample, which defines older adults as persons aged 50 and older, respondents in the SHARE survey were a very heterogeneous group.

Finally, it is also important to mention that data collection was carried out in an early stage of the pandemic. Thus, our study refers to a rather short-term impact of NPIs on mental health, which might result in an underestimation of the associations between government measures and mental health. In addition, the impact of a pandemic outbreak on mental health is often influenced by many different factors. For instance, we could not include measures such as job security, loneliness, or other psycho-social factors. Nonetheless, one of the strengths of this study is the combination of a large dataset on an individual level combined with very well-prepared data on a macro level from other sources, which allowed us to gain more specific insights into the complex associations of social factors, infection rates, government response, and decline in mental health.

## Conclusions

A majority of European older adults, who reported depressive symptoms or being nervous and/or anxious, showed a decline in mental health since the beginning of the COVID-19 pandemic. This holds especially true in countries with high prevalence rates of COVID-19. Among older European adults, age seems to be a protective factor for a decline in mental health while female gender apparently is a risk factor. Moreover, looking at government response measures, we conclude that, despite those NPIs being an essential preventative mechanism to reduce the pandemic spread, they might influence the vulnerability for elderly people suffering from mental health problems.

## Data Availability Statement

Publicly available datasets were analyzed in this study. This data can be found at: The SHARE data is available for research purpose after registering at the SHARE website (www.share-project.org). Data from the Oxford COVID-19 Government Response Tracker (OxCGRT) is freely available at https://github.com/OxCGRT/covid-policy-tracker. The COVID-19 Data Repository stores data on COVID cases and is located at https://github.com/CSSEGISandData/COVID-19. All websites were lastly accessed on 9 February 2022.

## Ethics Statement

The studies involving human participants were reviewed and approved by Ethics Council of the Max Planck Society. The patients/participants provided their written informed consent to participate in this study.

## Author Contributions

DL and OK developed the research questions. DL prepared, analyzed and interpreted the data, and drafted and finalized the manuscript. OK substantially contributed to interpreting the data, drafting the manuscript, and critically revised and approved the final manuscript. All authors contributed to the article and approved the submitted version.

## Funding

The SHARE data collection was primarily funded by the European Commission through FP5 (QLK6-CT-2001-00360), FP6 (SHARE-I3: RII-CT-2006-062193, COMPARE: CIT5-CT-2005-028857, SHARELIFE: CIT4-CT-2006-028812) and FP7 (SHARE-PREP: N°211909, SHARE-LEAP: N°227822, SHARE M4: N°261982). Additional funding from the German Ministry of Education and Research, the US National Institute on Aging (U01_AG09740-13S2, P01_AG005842, P01_AG08291, P30_AG12815, R21_AG025169, Y1-AG-4553-01, IAG_BSR06-11, OGHA_04-064) and from various national funding sources is gratefully acknowledged (see www.share-project.org, last accessed 27 December 2021).

## Conflict of Interest

The authors declare that the research was conducted in the absence of any commercial or financial relationships that could be construed as a potential conflict of interest.

## Publisher's Note

All claims expressed in this article are solely those of the authors and do not necessarily represent those of their affiliated organizations, or those of the publisher, the editors and the reviewers. Any product that may be evaluated in this article, or claim that may be made by its manufacturer, is not guaranteed or endorsed by the publisher.
